# Ubiquitin-protein ligase E3A (UBE3A) mediation of viral infection and human diseases

**DOI:** 10.1016/j.virusres.2023.199191

**Published:** 2023-08-04

**Authors:** Pankaj Chaudhary, Jessica Proulx, In-Woo Park

**Affiliations:** Department of Microbiology, Immunology and Genetics, School of Biomedical Sciences, University of North Texas Health Science Center, Fort Worth, TX 76107, United States

**Keywords:** UBE3A, E6-AP, HIV-1, HPV, HCV, Ubiquitin, Ubiquitin Proteasome System (UPS)

## Abstract

The Ubiquitin-protein ligase E3A, UBE3A, also known as E6-associated protein (E6-AP), is known to play an essential role in regulating the degradation of various proteins by transferring Ub from E2 Ub conjugating enzymes to the substrate proteins. Several studies indicate that UBE3A regulates the stabilities of key viral proteins in the virus-infected cells and, thereby, the infected virus-mediated diseases, even if it were reported that UBE3A participates in non-viral-related human diseases. Furthermore, mutations such as deletions and duplications in the maternally inherited gene in the brain cause human neurodevelopmental disorders such as Angelman syndrome (AS) and autism. It is also known that UBE3A functions as a transcriptional coactivator for the expression of steroid hormone receptors. These reports establish that UBE3A is distinguished by its multitudinous functions that are paramount to viral pathology and human diseases. This review is focused on molecular mechanisms for such intensive participation of UBE3A in disease formation and virus regulation.

## Highlights

• The Ubiquitin-protein ligase E3A, UBE3A, also known as E6-associated protein (E6-AP), plays an essential role in determination of the stability of various proteins, using the proteasomal degradation system.
• UBE3A decays key viral proteins in the virus-infected cells and, thereby, impacts on the infected virus life cycle followed by the viral diseases.
• Mutations or deletions in the maternally inherited gene in the brain cause human neurodevelopmental disorders such as Angelman syndrome and autism.

## 1 Introduction

Ubiquitination is a well-conserved post-translational modification process for attaching ubiquitin (Ub) or poly-Ub chains to substrate proteins to yield proteasomal degradation or changes in cellular signaling pathways; that is, the Ub proteasome system (UPS) plays an essential role in the regulation of protein homeostasis (Kleiger and Mayor, 2014; Martin-Villanueva et al., 2021; Stekel et al., 2022). The system consists of three sequential enzymatic actions: activation of a ubiquitin molecule by the E1 enzyme in an adenosine-triphosphate-dependent manner, leading to the formation of a covalent bond between the C-terminal end of Ub and a cysteine residue at the active site of E1; conjugation of the activated Ub to the active site of E2 enzyme; and the final transfer of the conjugated Ub with E2 to the substrate protein by E3 ligase. Transfer of Ub to the substrate protein is achieved in two different ways. RING class E3 ligases directly transfer Ub conjugated to E2 to the substrate, while homologous to the E6-AP carboxyl terminus (HECT) type E3 ligases transfer Ub from E2 to E3 ligases for the final transfer to the substrate protein.

UBE3A gene encodes a member of the HECT type E3 Ub ligase, UBE3A (E6-AP, E6-associated protein) of approximately 100 kDa (Huibregtse et al., 1993). The encoded UBE3A protein catalyzes the attachment of K48-linked Ub chains to its substrates, consequently targeting them for proteasomal degradation (Scheffner et al., 1993; Wang and Pickart, 2005). While there are several types of K-linked ubiquitination (K6, K48, K63 etc.), UBE3A carries out specifically K48-linked Ub chains (Kim and Huibregtse, 2009; Wang and Pickart, 2005). Therefore, UBE3A is one of the few E3 ligases with very specific modification pattern. UBE3A was originally named E6-associated protein (E6-AP) since the protein interacts with the E6 protein expressed by infected human papillomavirus (HPV), which causes cervical cancer, other anogenital cancers, and oropharyngeal cancers (Huibregtse et al., 1991; Munoz et al., 2003; Schiffman et al., 2005). The E6 protein interacting with UBE3A recruits the tumor suppressor p53 protein to form a ternary complex, resulting in p53 ubiquitination followed by proteasomal degradation, a crucial step in HPV-mediated carcinogenesis; that is, UBE3A-mediated degradation of p53 requires the E6 viral protein (Cooper et al., 2003; Huibregtse et al., 1993; LaSalle et al., 2015). Recent reports also indicated that UBE3A plays an integral part in regulating viral diseases, such as hepatitis C virus (HCV) (Kwak et al., 2016; Lee et al., 2022; Li et al., 2016; Munakata et al., 2007) and human immunodeficiency virus type I (HIV-1) (Ali et al., 2020; Pyeon et al., 2019), by destabilizing proteins encoded by viruses.

Besides viral diseases, mutations in the UBE3A gene cause human diseases, such as a rare neurodevelopmental disorder, Angelman syndrome (AS), and some cases of autism spectrum disorder (Kuhnle et al., 2018; Yamamoto et al., 1997). For example, mutation of the UBE3A gene in the maternally inherited chromosome 15 (15q11.2 - q13) induces AS (Albrecht et al., 1997; Buiting et al., 2016; Gustin et al., 2010; Judson et al., 2014; Sell and Margolis, 2015; Tan et al., 2014; Williams et al., 2010), and duplication of the gene is known to be associated with autism (Noor et al., 2015; Urraca et al., 2013; Yi et al., 2015). Therefore, this review is focused on the role of UBE3A in human and viral diseases and detailed molecular processes of UBE3A's mediation of those diseases.

## 2 Overview of UBE3A

**Fig. 1:**
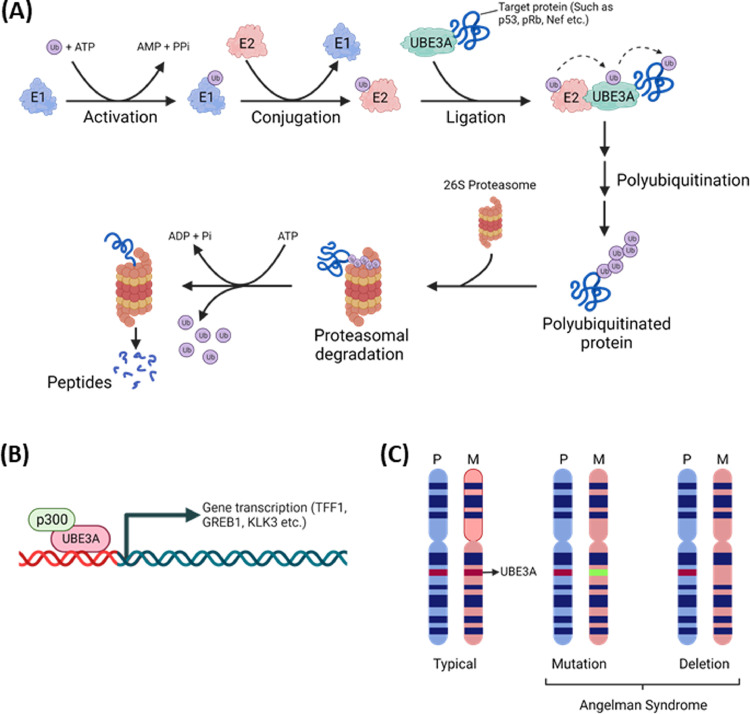
Overview of UBE3A functions. (A) Schematic representation of polyubiquitination process by UBE3A ligase containing HECT domain. The E1 ubiquitin-activating enzyme activates ubiquitin, then transfers to the E2 ubiquitin-conjugating enzyme and a HECT UBE3A ligase enzyme. The UBE3A ligase enzyme transfers the ubiquitin to the target protein, leading to its polyubiquitination. Known UBE3A targets include p53, pRb, Nef, etc. Polyubiquitinated protein is degraded to short peptides by the 26S proteasome. Ub, ubiquitin; E1, ubiquitin-activating enzyme; E2, ubiquitin-conjugating enzyme; E3, ubiquitin ligase. (B) UBE3A forms a complex with p300 and functions as a transcriptional regulator of genes such as TFF1, GREB1, KLK3, etc. Image created with biorender.com. (C) The UBE3A gene is located on chromosome 15 within the region of 15q11–15q13. Usually, only the maternal copy is expressed, while the paternal copy is silenced in neurons. In the case of AS, the maternal UBE3A allele is deleted or mutated.

The UBE3A protein is the family of E3 ligase proteins, containing the active site of the Ub ligase at its C-terminal domain known as the HECT domain, which transfers Ub from the E2 conjugation enzyme to the substrate (Kee and Huibregtse, 2007; Scheffner and Staub, 2007) (Fig. 1A). In the process of the Ub transfer, UBE3A simultaneously associates with the substrate protein and the E2 conjugation enzyme, preferentially ubiquitin-conjugating enzyme H7 (UbcH7) (Ronchi et al., 2013), albeit associating with other E2 conjugation enzymes like ubiquitin-conjugating enzyme H8 (UbcH8) via two E2 Ub binding sites (Ronchi et al., 2013), These differential interactions of UBE3A with the E2 conjugation enzyme may alter the specificity of the substrate selection (Eletr and Kuhlman, 2007). Since the interaction of UBE3A with the substrate proteins for the Ub transfer from the E2 conjugation enzyme is transient, even if UBE3A helps identify the substrates, identification of all substrate proteins, leading to UBE3A disease phenotypes (LaSalle et al., 2015), is difficult.

Transcription of the UBE3A gene generates three different isoforms, isoforms 1 to 3, by alternative splicing in human to encode three isoforms of UBE3A with distinct N-termini (RefSeq: NCBI. www.ncbi.nlm.nih.gov/refseq; GeneCards, The Human Gene Database, www.genecards.org). In mice, the transcript of UBE3A isoform 1 is truncated using an alternative polyadenylation site before the E3 ligase coding exon, while the N-terminus of isoform 1 is identical to isoform 3, equivalent to human isoform 3 (LaSalle et al., 2015). The isoform 1 generated by the alternative polyadenylation in mice, resulting in a noncoding isoform of UBE3A, is known to promote dendritic growth and spine maturation by acting as competing endogenous RNA to miR-134 (Valluy et al., 2015). The isoform 3 in mice and the corresponding isoform 1 in humans are regulated in early postnatal life, and UBE3A is predominantly stained in mature neurons, suggesting that the alternative splicing of UBE3A is critical in early life (Miao et al., 2013). The encoded UBE3A protein is located in both cytoplasm and nucleus and identified in the pre- and post-synaptic neuronal compartments (Dindot et al., 2008; Yashiro et al., 2009). Expression of UBE3A in both cytoplasm and nucleus is critical for the multifarious function of UBE3A, such as proteasome function, Wnt signaling, synaptic function, etc. (Lopez et al., 2018), and for the degradation of viral proteins located in both cytoplasm and nucleus, as described below. The synaptic localization of UBE3A is known to regulate the experience-dependent synaptic plasticity (Yashiro et al., 2009). Further, transferring the dual-isoform of human UBE3A improved motor learning and the execution of innate behaviors and mitigated exaggerated epileptogenesis and associated hippocampal pathology in AS mice (Judson et al., 2021). These reports indicate that alternative splicing to generate isoforms of UBE3A confers differential subcellular location of the UBE3A isoform and amelioration of AS.

UBE3A interacts with numerous cellular target proteins to exert its diverse biological functions. For the transfer of Ub from E2 conjugating enzymes to substrates to be degraded by the UPS, UBE3A associates with E2 conjugating enzymes, UBE2L3, UBE2D1, UBE2D2, and UBE2D3 (Jacobson et al., 2014). UBE3A, another E3 Ub ligase with a HECT domain family member, interacts with UbcH5 and UbcH7 (Schwarz et al., 1998). UBE3A also binds to and regulates protein homeostasis through Rpn10/PSMD4, a non-ATPase subunit of the 26S proteasome complex, which affects the stability of many proteins regulated by UBE3A (Jacobson et al., 2014; Lee et al., 2014; Wang et al., 2007). UBE3A is also known to interact with HERC2 physically (Galligan et al., 2015; Jacobson et al., 2014), and HERC2 interaction through the RCC1-like domain 2 of HERC2 and a region spanning amino acid residues 150–200 of UBE3A enhances the Ub-protein ligase activity of UBE3A (Kuhnle et al., 2011). All these interactions are known to impact the regulated degradation of many proteins by UBE3A profoundly.

UBE3A also functions as a transcriptional regulator of steroid hormone receptors, such as progesterone, estrogen, etc., by binding to the transcription complex (Catoe and Nawaz, 2011; Nawaz et al., 1999a, 1999b). Specifically, UBE3A forms a complex with p300 and other chromatin modifying enzymes at target gene promoters to create a transcriptionally active promoter environment (Catoe and Nawaz, 2011), and target genes activated by UBE3A include estrogen receptor responsive genes, trefoil factor 1 (TFF1) and growth regulating estrogen receptor binding 1 (GREB1) (Catoe and Nawaz, 2011), the androgen receptor responsive gene, kallikrein-related peptidase 3 (KLK3) (Khan et al., 2006) (Fig. 1B). According to Khan et al., UBE3A interacts with the androgen receptor (AR) and modulates both the protein level and the activity of AR, which is important for the growth and development of the prostate gland by regulating AR function as well as p53-mediated apoptosis (Khan et al., 2006). UBE3A is also known to form a complex with the nuclear estrogen receptor, ESR2, and degrade it, while the association of N-myc downstream-regulated gene 2 (NDRG2) with UBE3A prohibits UBE3A-mediated degradation of ESR2 (Zhu et al., 2020). Since ESR2 and NDRG2 are markedly low in colorectal cancer, enhancing ESR2 by blocking UBE3A-mediated degradation of ESR2 could offer a therapeutic target for CRC (Zhu et al., 2020).

Besides these reports, it is known that UBE3A targets cellular and viral proteins responsible for the severe neurodevelopmental disorder, such as AS and autism spectrum disorder (ASD), and for the progression of viral diseases caused by the infection of hepatitis C (HCV), human papilloma virus (HPV), and human immunodeficiency virus type 1 (HIV-1), as described below. Collectively, these reports indicate that UBE3A function is multifarious and imperative to regulating various human and viral diseases.

## 3 UBE3A in Angelman syndrome (AS) and autism spectrum disorder (ASD)

AS is a rare neuro-genetic disorder characterized by severe developmental delay, motor dysfunction, penetrant epilepsy, severe intellectual deficit, speech impairment, etc., (Buiting et al., 2016; Sell and Margolis, 2015), and the syndrome shares symptoms of autism, cerebral palsy, and Prader-Willi syndrome (Tan et al., 2014; Williams et al., 2010). AS is known to affect approximately 1 in 12,000 to 20,000 individuals with a 5–7 Mb deletion of the maternally inherited chromosome 15(15q11.2 - q13.3) in mature neurons (Buiting et al., 2016), while biallelically expressed in most peripheral tissues, in glia, and in newly born neurons (Albrecht et al., 1997; Gustin et al., 2010; Judson et al., 2014). That is, it is apparent that mutations in UBE3A within the region of the maternal chromosome are sufficient for causing AS, even if there were multiple genes in the region (Kishino et al., 1997; Matsuura et al., 1997). Furthermore, the severity of AS is related to the type of mutations in the region – the complete deletion of the region shows as the most severe, and point mutations in UBE3A as less severe (Gentile et al., 2010; Valente et al., 2013) (Fig. 1C). That is, disease severity can be modified with mutations of the additional genes within the 15q11.2-q13.3 locus, even if UBE3A were the predominant gene responsible for AS. It is reported that 1–2% of all autism spectrum disorder (ASD) contain a duplication of the chromosomal region (Cook et al., 1997; Sutcliffe et al., 1997), and the duplications of the region having only UBE3A are associated with developmental delay (Noor et al., 2015).

In addition to genotype-phenotype correlations between UBE3A and AS, the essentiality of UBE3A has been further proven. To elaborate, the downregulation of UBE3A expression using its specific shRNA inhibited apical dendrite outgrowth and impaired dendrite polarity. However, in mice, these impacts were obliterated by co-expression of UBE3A isoform 2 but not 1 or 3 (Miao et al., 2013). Further, deletion of the neuron-specific UBE3A was sufficient to induce AS-like phenotypes in mice experiments (Judson et al., 2016). Reinstatement of the gene using an adeno-associated virus-mediated transfer technique in neurons can prevent or mitigate AS symptomology in AS model mice (Judson et al., 2021, 2016; Silva-Santos et al., 2015) and neurons derived from patients with AS-like disease (Fink et al., 2017), demonstrating the significance of UBE3A in AS and therapeutic potential for human UBE3A gene transfer in the treatment of AS.

## 4 UBE3A in viral diseases

### 4.1 Hepatitis C Virus (HCV)

**Fig. 2:**
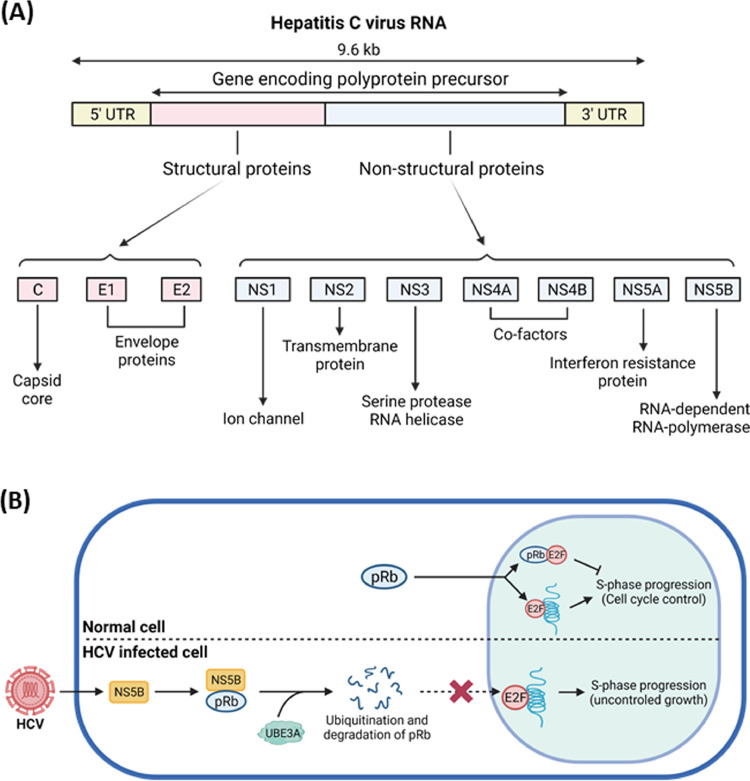
Role of UBE3A in HCV pathogenesis. (A) Schematic representation of the HCV genome. The HCV genome comprises an open reading frame (ORF) flanked by 5′ and 3′ untranslated regions (UTRs). Translation of the HCV ORF leads to the formation of a polyprotein that is further processed into three structural and seven non-structural viral proteins. The putative functions of the viral proteins are shown. (B) NS5B protein of the HCV virus forms a complex with the retinoblastoma tumor suppressor protein (pRb), thereby targeting it for UBE3A-mediated ubiquitination and proteasome-mediated degradation. This results in the release of the E2F transcription factor and activation of E2F-responsive promoters. Likewise, transcription of S-phase genes, G1- to S-phase progression and cell proliferation are stimulated. Image created with biorender.com.

HCV, which persistently infects the human liver, is an enveloped, positive-strand RNA virus belonging to the genus *Hepacivirus* of the family *Flaviviridae*. Chronic infection of HCV develops hepatitis, cirrhosis, and hepatocellular carcinoma (HCC), which has increased dramatically in recent years (Farazi and DePinho, 2006; Levrero, 2006), being a leading cause of morbidity and mortality for liver diseases worldwide (Pawlotsky, 2006). Entry of the virus requires key host factors, such as CD81 (Pileri et al., 1998), scavenger receptor type B class 1 protein (SRB-1) (Scarselli et al., 2002), Claudin-1 (Evans et al., 2007), Occludin (Liu et al., 2013; Ploss et al., 2009), and the recently identified SEC14L2 (Saeed et al., 2015), and the entered virus encodes a polyprotein from 9.6 kb genome which is processed to generate structural (core, E1, and E2) and non-structural (NS) proteins (NS1, NS2, NS3, NS4A, NS4B, NS5A, and NS5B) by both cellular and viral proteases (Fraser and Doudna, 2007) (Fig. 2A).

These expressed viral proteins are integral to developing HCV-mediated liver disease, even if inflammation associated with chronic HCV infection was known to be a critical culprit for HCC. To elaborate, HCV core protein, a nucleocapsid component, induces apoptosis and regulates the cell cycle of infected cells (Honda et al., 2000), and core, NS3, NS5A, and NS5B modulate cell proliferation independently from p53 expression in hepatocarcinoma cell lines (Siavoshian et al., 2004). Further, NS3 with a serine protease/helicase activity represses p21 (waf1) by impacting p53-dependent transcription activity (Kwun et al., 2001). It is critical for the innate host immune response via proteolytically inactivating various host cell factors that block viral replication. NS5A also regulates p21 (waf1) gene expression in a p53-dependent manner by associating with p53 (Majumder et al., 2001), and NS5B, an RNA-dependent RNA polymerase, down-regulates expression of the retinoblastoma tumor suppressor (Munakata et al., 2005). Previous reports indicated that the UPS restricted HCV replication by downregulating the levels of these key viral proteins, such as core, E2, NS1, NS2, NS5A, and NS5B (Gao et al., 2003; Haqshenas, 2013; Hou et al., 2010; Pavio et al., 2002; Suzuki et al., 2001), and stabilities of some HCV viral proteins are determined by UBE3A, indicative of the significance of the UPS-mediated degradation of these viral proteins in HCV pathogenesis.

Specifically, all-trans retinoic acid downregulates both protein and enzyme activity levels of DNA methyltransferase 1 and 3b and activates UBE3A expression through promoter hypomethylation (Lee et al., 2022). The enhanced UBE3A induces ubiquitination followed by degradation of the HCV core, impeding assembly to produce infectious HCV particles (Lee et al., 2022; Shirakura et al., 2007). Counteractively, the HCV core evades the Ub-dependent proteasomal degradation by inhibiting UBE3A expression via promoter hypermethylation (Kwak et al., 2016), which stimulates virus propagation. The stability of another structural viral protein, E2, an envelope protein, is also known to be regulated by UBE3A. In brief, adaptor protein complex 1 sigma 3 subunit (AP1S3), which enhances the production of HCV progeny virus particles, interacts with HCV E2 protein and thereby protects E2 protein from Ub-mediated proteasomal degradation (Li et al., 2016). UBE3A also associates with E2 protein, and blocking the interaction between AP1S3 and E2 with a synthetic peptide containing the AP1S3-recognizing motif inhibited HCV infection (Li et al., 2016), indicating that AP1S3 plays an important role in the regulation of UBE3A-mediated degradation of E2. UBE3A also plays an essential role in regulating the stability of the retinoblastoma (pRb) tumor suppressor protein in a NS5B-dependent manner (Munakata et al., 2007). NS5B traps pRb in the cytoplasm and recruits UBE3A to NS5B and pRb complex for UBE3A-mediated degradation of pRb (Munakata et al., 2007) (Fig. 2B), which promotes hepatocellular proliferation for the development of liver cancer. These reports indicate that the interactions, followed by reciprocal degradation regulation between UBE3A and HCV viral proteins, are critical for regulating the progression of HCV-mediated HCC.

### 4.2 Human immunodeficiency virus type 1 (HIV-1)

**Fig. 3:**
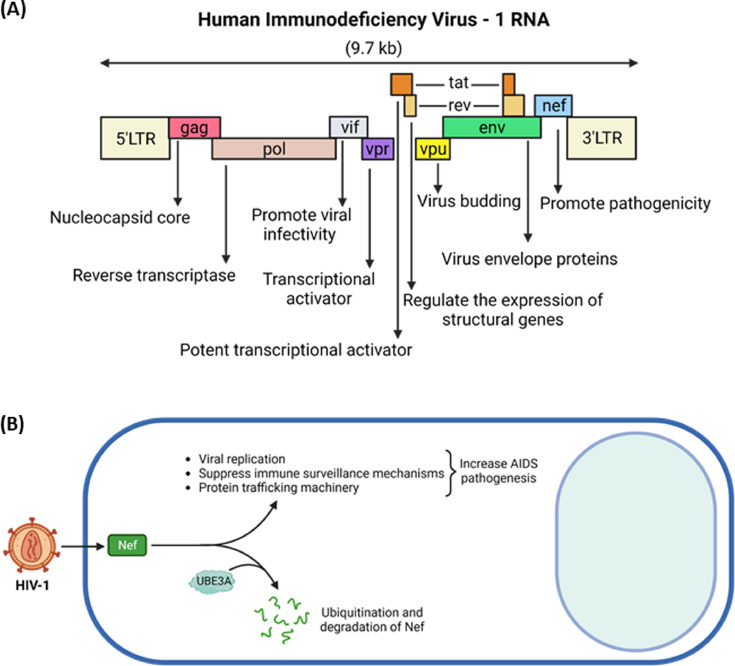
Role of UBE3A in dysregulation of HIV-1 pathogenesis. (A) Schematic representation of the HIV-1 genome. The total size of the HIV-1 genome is approximately 9.7 kb and is flanked by two long terminal repeats (LTR) promoter regions with nine open reading frames, which encode Gag, Pol, and Env polyproteins, four accessory proteins (Vpu, Vif, Vpr, Nef) and two regulatory proteins (Rev, Tat). The putative functions of the viral proteins are shown. (B) The key pathogenic HIV-1 viral protein, Nef, interacts with UBE3A ligase, leading to ubiquitination and proteasome-mediated degradation of Nef. The interaction between Nef and UBE3A is integral to regulating viral and cellular protein decay and, thereby, the competing HIV-1 and host cell survivals. Image created with biorender.com.

HIV-1 harboring a pair of non-covalently linked positive sense RNA enclosed by a conical capsid composed of the viral protein, p24, (Lu et al., 2011) requires interactions of HIV-1 envelope (Env) with the cellular receptor (CD4) and coreceptors (CXCR4 and CCR5, depending on the target cells) to infect the target cells (Berger et al., 1998; Keele et al., 2008; Kwong et al., 1998; Margolis and Shattock, 2006). The infecting virus replicates in the host cells, encoding at least six regulatory proteins, Vif, Vpr, Vpu, Tat, Rev, and Nef, in addition to three structural proteins, Gag, Pol, and Env, which are present in all standard retroviruses, in a stage-specific manner (Frankel and Young, 1998; Li et al., 2015) (Fig. 3A). That is, the early regulatory proteins, such as Tat, Rev, and Nef, are expressed at the early stage, and the expressed Rev transits the expression of viral proteins from the early to the late stage by exporting messages comprising the Res-responsive element (RRE) to the cytoplasm so that structural proteins are produced (Arrigo and Chen, 1991; Daly et al., 1989; Emerman et al., 1989; Feinberg et al., 1986; Heaphy et al., 1990; Malim and Cullen, 1991). Therefore, for the smooth transitioning of the HIV-1 life cycle from early to late viral gene expression, the stage-specifically expressed proteins should be degraded in a stage-specific and coordinated manner. In fact, viral proteins in HIV-1-infected cells exploit cellular machinery to decay viral and cellular proteins at every step of the HIV-1 life cycle: Nef/Env/Vpu degrade CD4 critical for the virus entry and superinfection (Aiken et al., 1994; Garcia and Miller, 1992; Lama et al., 1999; Levesque et al., 2004; Mariani and Skowronski, 1993; Ruiz et al., 2010; Wildum et al., 2006; Willey et al., 1992), The tripartite motif-containing protein 5α (TRIM5α)-mediated degradation of Gag is integral to species tropism (Arriagada et al., 2011; Battivelli et al., 2010; Chatterji et al., 2006; Sakuma et al., 2007; Shi and Aiken, 2006; Zhang et al., 2008), Vif/APOBEC3G determines the susceptibility of HIV-1 (Desimmie et al., 2014; Ehrlich and Yu, 2006; Mangeat et al., 2003; Mariani et al., 2003; Stopak et al., 2003; Yu et al., 2003), c-Cbl interacts with Nef and decays it (Zhang et al., 2020), and Vpu-triggered degradation of Tetherin is essential for release of the assembled virus particles (Harris et al., 2012; McNatt et al., 2009; Miyagi et al., 2009; Neil et al., 2008), etc. Further, it is reported that HIV-1 Nef plays an important role in regulating the stability of another key HIV-1 regulatory protein, Tat (Sugiyama et al., 2011).

However, the role of UBE3A in the regulation of the stability of HIV-1 viral proteins and its consequences on dysregulation of HIV-1 life cycle and pathogenesis is unknown or still in the nascent stage such that PubMed search lists only three publications associated with UBE3A and HIV-1, indicating that UBE3A may not regulate many viral and cellular proteins in HIV-1 infected cells or many HIV-1 viral proteins whose stability is regulated by UBE3A. Pyeon et al. showed that UBE3A interacted with HIV-1 Nef and degraded it (Pyeon et al., 2019). Further, UBE3A reduced the level of HIV-1 structural protein, Gag, only in the presence of Nef (Pyeon et al., 2019). These data and the report that Nef degrades Tat (Pyeon et al., 2019; Sugiyama et al., 2011) indicate that Nef and UBE3A complex plays a crucial role in coordinating viral protein degradation and hence HIV-1 replication, providing insights into the nature of pathobiological and defense strategies of HIV-1 and HIV-infected host cells (Fig. 3B).

Another report also demonstrated that UBE3A and HIV-1 Nef interacted in both Nef-transfected HEK-293T cells and HIV-1-infected MOLT3 cells, and these interactions are critical for enhancing p53 ubiquitination and degradation (Ali et al., 2020). Specifically, Nef enhanced p53 degradation, and UBE3A, but not its dominant negative mutant, further augmented Nef-mediated ubiquitination followed by degradation of p53 to reduce p53-associated apoptosis (Ali et al., 2020). Recent reports indicated that HIV-1 Nef is necessary and sufficient for cell activation and HIV-1 replication in the quiescent CD4+ T lymphocytes, indicating that Nef contributes to the establishment of HIV-1 latency (Arenaccio et al., 2014). It is also reported that Nef favors HIV-1 replication in the quiescent CD4+ T lymphocytes by modulation of TCR signaling cascade (Neri et al., 2011). Further investigation is needed to elucidate molecular mechanisms of UBE3A-mediated degradation of Nef for HIV-1 latency.

These data indicate that interaction between Nef and UBE3A is important for the cooperative ubiquitination and degradation of p53. Finally, it is reported that UBE3A shares an epitope with HIV-1 Env recognized by four CD4 binding sites, thus playing an important role in determining the neutralization breadth (Liu et al., 2015). According to the report (Liu et al., 2015), four (VRC01, VRC02, CH106, and CH103) of nine broadly neutralizing antibodies (bNAbs) specific for the HIV-1 CD4 binding site bind UBE3A. Thus, UBE3A competitively inhibits the binding of gp120 to the VRC01 bNAbs, regulating the neutralization breadth. These reports indicate that UBE3A impacts HIV-1-associated pathogenicity by regulating apoptosis of HIV-1-infected cells, replication of HIV-1, and antibody reactivities in HIV-1-infected patients.

### 4.3 Human papilloma virus (HPV)

In contrast to HIV-1 and HCV, HPV is a non-enveloped icosahedral, circular, double-strand (ds) DNA virus belonging to the *Papillomaviridae* family (Ljubojevic and Skerlev, 2014). It is reported that more than 207 HPVs have been classified (Van Doorslaer et al., 2017), and these viruses are placed into five groups (α, β, ɣ, μ, and ν) based on their genotype (Benevolo et al., 2016; Graham, 2017). Of these, the α group mainly infecting mucosal epithelia is the largest one, including approximately 15 so-called high-risk (HR) types (HPVs 16, 18, 31, 33, etc.) (Stanley, 2010) which cause cervical cancer and other anogenital preneoplastic diseases and cancers, such as vulvar, vaginal, and anal cancers in women, and anal and penile cancers in men (Anic and Giuliano, 2011; Benevolo et al., 2016; Giuliano et al., 2015; Graham, 2017). The β group, mainly infecting cutaneous epithelia, is the next largest group (Bzhalava et al., 2014; Kocjan et al., 2015; Van Doorslaer, 2013) and is associated with non-melanoma squamous cell carcinomas, the most common human cancer (Tommasino, 2017). The remaining groups (ɣ, μ, and ν) cause only benign disease (Graham, 2017). In addition, HPV is also associated with several other cancers, such as prostate, colon, bladder, esophageal, and breast cancers (Heidegger et al., 2015). Among these oncogenic HPVs, most research has focused on HPV16, which is the cause of approximately 55% of cervical cancer, and HPV18, responsible for about 15% of cases (Stanley, 2010).

**Fig. 4:**
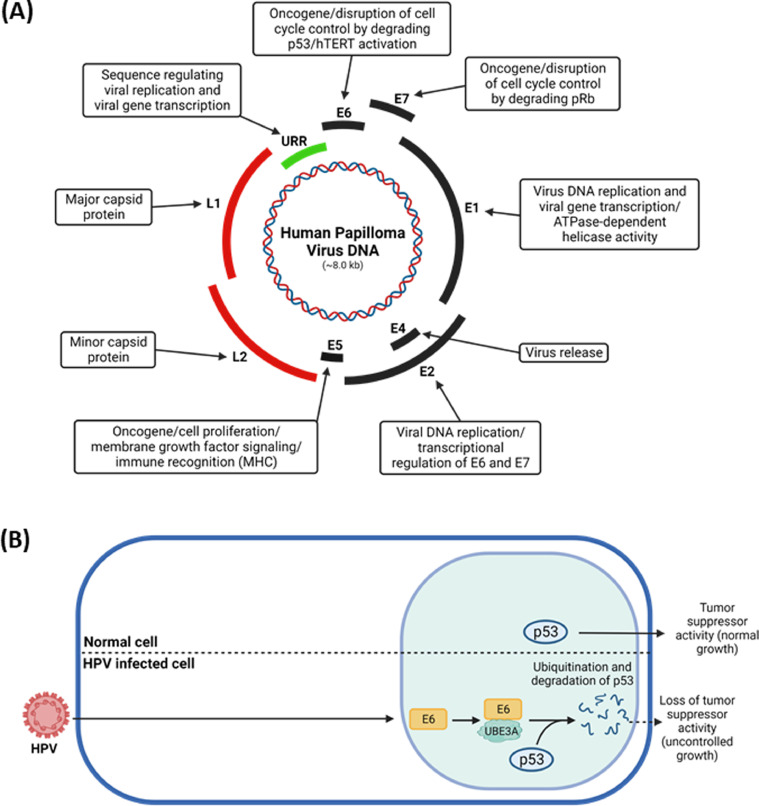
Role of UBE3A in HPV pathogenesis. (A) Schematic representation of the HPV genome. HPV genome consists of approximately 8 kb circular double-stranded DNA and is divided into early, late, and non-coding upstream regulatory regions (URR). The early genome encodes E1, E2, E4, E5, E6, and E7 regulatory proteins, and the late genome encodes L1 and L2 capsid proteins. The putative functions of the viral proteins are shown. (B) The E6 oncoprotein of the HPV virus interacts with UBE3A ligase and causes the ubiquitination and proteasome-mediated degradation of the tumor suppressor p53, therefore, leading to cancer malignancies. Image created with biorender.com.

All HPVs harbor an episomal DNA genome which is divided into three functional sections; the early (E) region encoding at least six regulatory proteins (E1, E2, E4, E5, E6, and E7), the late (L) region expressing two structural proteins (L1 and L2), and the long control region (LCR; also known as the upstream regulatory region (URR)) containing the viral *cis*-acting regulatory sequences that control viral replication, transcription, and post-transcriptional control through the late regulatory element (LRE) (Graham, 2010) (Fig. 4A). Once the virus infects the dividing epithelial cells where its episomal dsDNA enters the nucleus, the infected HPV encodes the viral replication/transcription factors E1 and E2 (Ozbun, 2002). The encoded E1 and E2 transcription factor regulates the early viral promoter to direct expression of the key oncoproteins, E6 and E7, which contribute to forming the papilloma-regulatory proteins that ensure the survival of HPV in the infected cells (Bernard, 2013; Moody and Laimins, 2010; Romanczuk and Howley, 1992; Schwartz, 2013; Thierry, 2009).

UBE3A interacts with the E6 oncoprotein of HPV16, which causes anogenital cancers, most notably cancer of the uterine cervix, via the HECT domain (Gu et al., 2001; Huibregtse et al., 1993; Sailer et al., 2018; Scheffner et al., 1993; Schwarz et al., 1998; Talis et al., 1998; zur Hausen, 2002), while E7 oncoprotein in association with cullin Ub ligases and UBR4 interacts with retinoblastoma (Rb) family of tumor suppressors, resulting in Ub-mediated targeted degradation of the associated Rb family (DeMasi et al., 2005; Huh et al., 2007, 2005; McLaughlin-Drubin and Munger, 2009) In the UBE3A/E6 complex, E6 recruits p53 and the transfer of Ub from a thioester cysteine bond in the UBE3A ubiquitination domain to p53 tumor suppressor protein and subsequent degradation of p53 (Gu et al., 2001; Scheffner et al., 1993) (Fig. 4B), contributing to the progression of HPV-associated tumorigenesis. In the absence of E6 in the complex, UBE3A does not target p53 and other proteins for ubiquitination and degradation by the 26S proteasome (Beaudenon and Huibregtse, 2008; Scheffner et al., 1993; Scheffner and Whitaker, 2003), 151]. In this process, two different modes of interactions between UBE3A and E6 have been identified with diverse human and non-human papillomaviruses E6 (Drews et al., 2020). First, in type I interactions, the LXXLL motif alone of UBE3A interacts with E6 and E6 in the complex recruit cellular substrates, such as p53 (Chen et al., 1998; Drews et al., 2020; Elston et al., 1998). In type II interactions, besides the LXXLL motif, the additional auxiliary regions of UBE3A in either the amino terminus or the carboxy-terminal HECT domain are required to interact with the LXXLL motif of UBE3A (Drews et al., 2020). The amino-terminal UBE3A not only enhances the association of LXXLL with E6 but also requires E6 to trigger Ub ligase activity in the carboxy-terminal HECT Ub ligase domain of UBE3A (Drews et al., 2020). This type II interaction is needed for the degradation of p53 or Na^+^/*H*^+^ exchanger regulatory factor 1 (NHERF1), and this type-specific interaction provides vital information for aiming to interfere with the activity of the UBE3A/E6 complex.

## 5 Discussion and prospects of therapeutics

As reviewed above, the UBE3A function is multifarious and critical in regulating of various human and viral diseases. It is responsible for severe neurodevelopmental disorders, such as AS and ASD, due to the mutation of the *UBE3A* gene and, thereby, loss of UBR3A function and could be involved in the regulation of the progression of viral diseases caused by HCV, HIV-1, and HPV by interacting with the viral protein of each virus. Molecular mechanisms on UBE-associated AS and HPV-medicated cancers are relatively well addressed. However, the roles of UBE3A in HCV- and HIV-1-triggered hepatocellular carcinoma and AIDS, respectively, are as yet unknown, and thus treatment of these diseases is still at its incipient stage.

Since the essentiality of UBE3A for AS is proven from the finding that deletion of neuron-specific UBE3A is proficient in the induction of AS-like phenotypes in mice, a leading approach for developing therapeutics is focused on reinstatement of the gene through gene therapy techniques or nonsilencing paternal UBE3A expression. Specifically, adeno-associated virus- (AAV-) mediated transfer of the functional UBE3A has been successfully reinstated for the functional, long-lasting UBE3A expression in neurons (Li and Samulski, 2020; Tsagkaris et al., 2020), yielding promising signs for the treatment of AS via gene therapy. Furthermore, it was reported that AAV-mediated transfer of UBE3A successfully rescued the cognitive defects in a mouse model for AS (Daily et al., 2011). Moreover, the safety and effectiveness of AAV-associated gene transfer for treating central nervous system (CNS) disorders have been approved by Food and Drug Administration (FDA) (Mendell et al., 2017; Moore et al., 2018). Therefore, more frequent trials to apply this technology toward treating AS could be inferred. Reinstatement of UBE3A in neurons has been achieved by using topoisomerase inhibitors (Huang et al., 2011), targeting a long non-coding RNA (Meng et al., 2015; Wolter et al., 2020), or CRISPR/Cas9 (Bailus et al., 2016; Schmid et al., 2021; Wolter et al., 2020) that restore the expression of UBE3A.

In the virus-infected cells, UBE3A interacts specifically with viral proteins and degrades the interacting viral proteins in HCV and HIV-1 or recruits and degrades tumor suppressor molecule, p53, in HPV-infected cells, as described above. Recently, targeted protein degradation (TPD) has emerged as a therapeutic modality to tackle critical pathogenic proteins, using proteolysis-targeting chimera (PROTAC) protein degraders, also known as molecular glues (Bekes et al., 2022; Cruz Walma et al., 2022; Schapira et al., 2019). The PROTAC degraders consist of two ligands joined by a linker: one ligand recruits and bind a protein of interest (POI), while the other recruits and binds an E3 Ub ligase. POI degradation by E3 Ub ligase is potent since the PROTAC degrader enables specific POI/E3-ligase interaction and can be reused in the degradation reaction (Bekes et al., 2022; Cruz Walma et al., 2022; Schapira et al., 2019). Interactions of UBE3A followed by degradation of the core (Lee et al., 2022; Shirakura et al., 2007) and envelope (Li et al., 2016) of HCV and Nef of HIV-1 (Ali et al., 2020; Pyeon et al., 2019) are perfect pairs of POI and E3 Ub ligase, and thus efficacious PROTAC degrader development for the decay of these viral proteins will lead directly to creating potently specific therapeutics to incapacitate HIV-1. PROTAC or its modified strategies are also being applied to incapacitate the POI by E3 Ub ligase in a different manner - UBE3A ligase modulators acting as molecular glues for altering the target protein-binding properties of UBE3A to promote interaction with proteins of therapeutic interest (Gandhi et al., 2014; Ito et al., 2010; Ito and Handa, 2016; Matyskiela et al., 2016) and ligand-directed degraders to connect via a linker designed to interact with the UBE3A and the target protein (Matyskiela et al., 2016); and PROTAC glue linking intracellularly expressed antibody directed against E6 to incapacitate E6 and thereby blocking UBE3A/E6 complex-mediated p53 ubiquitination followed by degradation, is also among the promising therapeutic designs (Amici et al., 2016; Griffin et al., 2006). Hence, these formulations and the collection of other studies described in the present review evince the many remarkable capacities of UBE3A impactful to viral infection, human disease, and the growing mechanistic comprehension enabling their ultimate treatments and prevention.

## Declaration of Competing Interest

The authors declare that they have no known competing financial interests or personal relationships that could have appeared to influence the work reported in this paper.

## Data Availability

Data will be made available on request.
